# Tanzanian lessons in using non-physician clinicians to scale up comprehensive emergency obstetric care in remote and rural areas

**DOI:** 10.1186/1478-4491-9-28

**Published:** 2011-11-09

**Authors:** Angelo S Nyamtema, Senga K Pemba, Godfrey Mbaruku, Fulgence D Rutasha, Jos van Roosmalen

**Affiliations:** 1 Tanzanian Training Centre for International Health, Ifakara, United Republic of Tanzania; 2 Department of Obstetrics & Gynaecology, St Francis Designated District Hospital, Ifakara, United Republic of Tanzania; 3 Ifakara Health Institute, Dar es Salaam, United Republic of Tanzania; 4 UNFPA Country Office, United Republic of Tanzania; 5 Department of Obstetrics, Leiden University Medical Centre, the Netherlands; 6 Department of Medical Humanities, EMGO-Institute for Health and Care Research, VU University Medical Centre Amsterdam, the Netherlands

## Abstract

**Background:**

With 15-30% met need for comprehensive emergency obstetrical care (CEmOC) and a 3% caesarean section rate, Tanzania needs to expand the number of facilities providing these services in more remote areas. Considering severe shortage of human resources for health in the country, currently operating at 32% of the required skilled workforce, an intensive three-month course was developed to train non-physician clinicians for remote health centres.

**Methods:**

Competency-based curricula for assistant medical officers' (AMOs) training in CEmOC, and for nurses, midwives and clinical officers in anaesthesia and operation theatre etiquette were developed and implemented in Ifakara, Tanzania. The required key competencies were identified, taught and objectively assessed. The training involved hands-on sessions, lectures and discussions. Participants were purposely selected in teams from remote health centres where CEmOC services were planned. Monthly supportive supervision after graduation was carried out in the upgraded health centres

**Results:**

A total of 43 care providers from 12 health centres located in 11 rural districts in Tanzania and 2 from Somalia were trained from June 2009 to April 2010. Of these 14 were AMOs trained in CEmOC and 31 nurse-midwives and clinical officers trained in anaesthesia. During training, participants performed 278 major obstetric surgeries, 141 manual removal of placenta and evacuation of incomplete and septic abortions, and 1161 anaesthetic procedures under supervision. The first 8 months after introduction of CEmOC services in 3 health centres resulted in 179 caesarean sections, a remarkable increase of institutional deliveries by up to 300%, decreased fresh stillbirth rate (OR: 0.4; 95% CI: 0.1-1.7) and reduced obstetric referrals (OR: 0.2; 95% CI: 0.1-0.4)). There were two maternal deaths, both arriving in a moribund condition.

**Conclusions:**

Tanzanian AMOs, clinical officers, and nurse-midwives can be trained as a team, in a three-month course, to provide effective CEmOC and anaesthesia in remote health centres.

## Background

In Tanzania, 47% of pregnant women deliver in health facilities and only 46% of deliveries are assisted by skilled personnel [1,2]. The met need for emergency obstetric care, at 15-30%, and the caesarean section rate (CSR) of 3% are still below ideal levels and constitute the lowest rates in the world [1,3]. The majority of these health facility deliveries and caesarean sections are for women in urban areas, where services are more accessible. Such low CSR indicates that a significant number of mothers is denied the service which is quite often a life-saving option for failed and/or high-risk vaginal delivery. The above figures can partly explain the unacceptably high maternal mortality ratio (449/100 000 live births) in the country [4]. This can be linked to the existing shortage of skilled staff and inadequate health facilities with comprehensive emergency obstetrical care (CEmOC).

The shortage of human resources for health in Tanzania is one of the most severe in Africa [3-6]. The available skilled workforce is only 32% of that recommended [5]. The Government of Tanzania began training assistant medical officers (AMOs) in the early 1960s. These are non-physician clinicians (NPCs) selected from a lesser-trained cadre of clinical officers (COs) for a 2-year programme, which includes three months of surgery and three months of obstetrics. They are meant to be general practitioners, but are licensed to perform major surgery independently, including caesarean section. There is no provision for internship, residency, or other formal post graduate training for AMOs. Most have done fewer than the required five caesarean sections at the time of graduation. The need for more hands-on experience is met by informal training with more experienced staff at the hospitals where they are sent to work. Usually AMOs do not operate independently until after six months on duty with other staff. Outside of cities, 85% of emergency obstetric surgery is performed by AMOs, both working in government and mission hospitals [6]. There are only 1600 doctors, mostly concentrated in the biggest cities, 2000 AMOs, 8000 COs and 15,000 nurse-midwives (NM) in the work force in Tanzania, a country with an estimated population of 40 million people [7].

Recently, the government of Tanzania revised the National Health Policy with a goal to improve the health and well being of all Tanzanians with a focus on those most at risk, and to encourage the health system to be more responsive to the needs of the people [8]. One of its strategies is to upgrade health centres and use NPCs to improve accessibility to CEmOC in remote rural areas where the majority (77%) of Tanzanians live [1,5]. It is with this background that we took up the challenge to develop and launch three months postgraduate training programmes for AMOs in CEmOC, and for CO and NM in anaesthesia. Our research questions were: does this three months training of AMOs in CEmOC better address workplace needs compared to current training, and can a three months comprehensive training of NM and COs in anaesthesia result in acceptable quality of care?

## Methods

### Settings

While there are seven AMO schools with an average annual output of 200 there is only one medical school in the country where graduate doctors are trained to specialize in anaesthesia. Currently, there are only 17 specialists in anaesthesia in the whole country. The majority (14) work in Dar es Salaam hospitals. There is one institution where AMOs specialize in anaesthesia and another one where NM and COs are trained as anaesthetic nurses (anaesthetic assistants). These AMO and nurse anaesthetists only partially relieve the shortage. To meet the need for the upgraded health centres, AMOs were trained in comprehensive emergency obstetrical care while COs and NMs, as anaesthetic assistants, were trained to give spinal anaesthesia and ketamine general anaesthesia. The trainees were recruited in teams which comprised of at least one AMO and two NMs or COs from the same facility. The concept of team training was devised in order to ensure inclusion of key categories of staff able to perform obstetric surgeries and anaesthesia.

### Training venue and capacity

The training took place in two collaborating institutions: Tanzanian Training Centre for International Health (TTCIH) and Saint Francis Designated District Hospital (SFDDH). TTCIH is a non profit semi-autonomous institution that offers short international courses in health and a long course for AMOs. The two institutions (TTCIH and SFDDH) have had long experiences in health related training and health care service delivery. SFDDH, a hospital with a 372-bed capacity, receives referred patients from primary health facilities (dispensaries and health centres) in Ulanga and Kilombero districts. The mean annual delivery and caesarean section rates from 2005 to 2008 were 4,987 and 25% respectively. The key technical staff for the programmes included one medical curriculum expert, two obstetricians, one paediatrician, two generalist doctors and one senior AMO - all with vast experience in maternal and perinatal care. The training in anaesthesia was conducted by a consultant anaesthetist from Muhimbili National Hospital (MNH), one AMO specialized in anaesthesia and two senior anaesthetic nurses from SFDDH. The training programmes were built on the framework of human resources, pedagogical and technological materials available in the two institutions.

### Teaching and learning processes

Competency-based training curricula for CEmOC and anaesthesia were developed. The process of curriculum development included: occupational profiling, assessment of the employers' needs in maternal health, clarification of objectives including required competencies, description of the methodology for implementation of the curricula, establishment of financial implications and documentation of the human and physical resources needed for effective learning and teaching.

The main emphasis of both training curricula included the underlying principles in obstetric and anaesthetic care; appropriate decision making and clinical reasoning skills, and acquisition of clinical management skills. The training in CEmOC required the trainees to attain the following key competencies by the end of the training:

• Ability to diagnose and manage uncomplicated labour and recognize complications arising during labour;

• Ability to determine when operative vaginal or abdominal delivery is indicated and be able to perform such procedures;

• Ability to diagnose and treat problems of the newborns (selected conditions).

The training programmes took three months and involved both hands-on and theory. All trainees for both (CEmOC and anaesthesia) programmes were included in night duty rosters in groups of two attached to more experienced hospital staff. The scope of working activities under supervision was outlined. The CEmOC trainees were also included in the day-time labour ward duty roster and were also involved in routine teaching ward rounds in the maternity which were carried out by the hospital obstetric team thrice a week. During these ward rounds and when they were on call, the CEmOC programme participants were included in the decision making for patients requiring surgical interventions. They were also involved in elective and emergency obstetric surgeries, either as assistant or operating independently. Elective obstetric surgeries were performed twice a week. Participants for the anaesthesia programme took part in all surgical, obstetric and gynaecological elective and emergency operations, either as assistant to a qualified anaesthetist or giving anaesthesia under supervision.

Demonstrations of procedures were made during actual performance as well as using available manikins and video films at TTCIH's Clinical Skills Laboratory with ample opportunity to practice these using the manikins. Procedures were supervised and candidates reached the level of proficiency before they were allowed to manage patients. These included resuscitation of the newborn, vacuum extraction, caesarean section, abdominal aorta compression and condom tamponade for management of postpartum haemorrhage and intubation. Interactive lectures were conducted on every working day (five days a week) for at least 2 hours, from 14:00 to 16:00. Teaching emphasis for AMOs was put on all elements of CEmOC; clinical presentations; diagnosis; complications; and treatment and prevention of complications of pregnancy and childbirth. Other areas included peri-operative care, resuscitation and infection prevention. The training in anaesthesia emphasized the use of spinal anaesthesia and ketamine, and covered a wide range of topics including classification, methods, indications, contraindications, potential complications and management. Various available anaesthetic drugs were discussed. Problems unique to anaesthesia in obstetrics - along with medical conditions related to obstetrics, including haemorrhage, anaemia, (pre) eclampsia and respiratory diseases - were dealt with. Other areas included resuscitation, oxygen therapy, peri-operative care, sterilization, infection prevention and operating room etiquette (scrubbing, masks, gloving and catheterization). Adult learning and teaching methods were encouraged to improve the learning processes for both programmes.

### Assessment of teaching and learning processes

Each trainee was given a logbook at the start of the training. Lists of obstetric and anaesthetic procedures were developed, and the minimum targets (numbers) required for each course participant were indicated in the logbooks. Procedures required for CEmOC programme participants included spontaneous vertex deliveries, assisted breech deliveries, repair of cervical and perineal tears, vacuum deliveries, caesarean sections, laparotomy for ruptured uterus (repair or subtotal hysterectomy), laparotomy for ruptured ectopic pregnancy, manual removal of placenta and evacuation of inevitable, evacuation of incomplete or septic abortions. Anaesthetic procedures included spinal anaesthesia, intubation of adults for general anaesthesia, administration of general anaesthesia using ketamine and resuscitation of newborns. All procedures performed by the trainees were documented in the logbooks and countersigned by their supervisors. Outcomes for mother and infant were recorded. All surgical procedures were also documented in the operating theatre record books.

End of course assessment was carried out using Objectively Structured Clinical Examinations (OSCE) as well as written examinations. In addition, the funder of the first batch contracted a team for mid-evaluation and gave feedback in writings to the course coordinator who further shared the findings with other facilitators. This evaluation involved interviews with the course coordinator, facilitators and participants on several occasions.

### Performance of upgraded health centres

The World Lung Foundation (WLF) upgraded CEmOC services in four health centres between March and June 2010. The first author of this paper was appointed by WLF to follow up the course by carrying out monthly supportive supervision and to report on the performance of the three upgraded health centres, located in Ulanga and Kilombero districts in Morogoro region, i.e. Mwaya, Mtimbira and Mlimba. During the visits, for 2-3 days in each health centre the team conducted training sessions in obstetric care, took part in management of in- and out-patients and reviewed data on obstetric care and outcome. Institutional maternal mortalities and fresh stillbirths were used as indicators for assessing the quality of obstetric outcome in these centres. Referred obstetric cases were also documented. The plan was to establish a supervisory system that will become less intensive, but will continue indefinitely from the district hospitals related to these health centers. The same procedure has been established in the two other regions served by the WLF program. Data was entered into excel and analyzed using Stata software.

## Results

### Number of trained NPCs

Three batches with a total of 45 participants for both programmes were trained from June 2009 to April 2010. The first batch had 10, second had 23 and third had 12 participants. Thirteen participants were sponsored by the World Lung Foundation through Ifakara Health Institute, 20 by UNFPA through the Ministry of Health, 10 by Lions Club International (Sweden) and two were participants from Somalia sponsored by Trocaire Somalia Programme. A total of 14 AMOs were trained in CEmOC and 31 (clinical officers and nurse/midwives) were trained in anaesthesia. Participants were trained in teams from 12 health centres located in Morogoro, Dodoma and Coastal regions, where the funders in collaboration with the respective 11 district health authorities had planned to extend CEmOC services. Of these health centres, 11 were located in rural districts which were as far as 150 km (Mlimba health centre) from the nearest referral hospital, to which they referred complicated obstetric cases. One CEmOC programme participant dropped out because of social problems and his performances were not included in this report.

### Performances of the course participants in the training centre

A total of 278 major obstetric surgeries (C-sections, laparotomies for ruptured uterus and ectopic pregnancies) were performed under supervision by the CEmOC trainees. On average each participant performed more than three quarters of the minimum targets for uncomplicated deliveries, caesarean sections, repair of cervical and perineal tears and evacuation of inevitable, incomplete and septic abortions. Because of the relatively small number of cases of ruptured uterus during the three months (even in a very busy district hospital), the participants were exposed to only 33% of the minimum targets for surgeries on ruptured uterus (Table 1).

**Table 1:** Proportions of obstetric procedures performed during training by Assistant Medical Officers trained in Comprehensive EmOC

Category of procedures	Total number of procedures performed	Minimum target set per course participant	Proportions performed per participant
Normal deliveries	207	15	107% (15)
Breech delivery assisted	35	5	60% (3)
Repair of cervical and perineal tears	111	11	81% (8)
Caesarean sections	208	15	107% (15)
Vacuum deliveries	20	5	40% (2)
Operation on ruptured uterus (repair or subtotal hysterectomy)	26	6	33% (2)
Laparotomy for ruptured ectopic pregnancy	44	7	43% (3)
Manual removal of placenta	38	5	60% (3)
Evacuation of inevitable, incomplete and septic abortions	103	10	80% (7)

A total of 1161 anaesthetic procedures were performed by the trainees in anaesthesia. On average each participant performed all (100% to 110%) minimum targets of procedures for spinal anaesthesia and administration of anaesthesia using bolus ketamine. However, there were very few patients who were operated using general anaesthesia who needed endotracheal intubation. In this case participants were exposed to as low as 23% of the minimum targets (Table 2). Anaesthetic assistants were also trained on how to resuscitate a newborn baby and how to assist the surgeon during operations.

**Table 2:** Proportions of anaesthetic procedures performed during the training by clinical officers and nurse-midwives trained in anaesthesia

Category of procedures	Total procedures performed	Minimum targeted per candidate	Proportions performed per candidate
Spinal anaesthesia	625	20	100% (20)
Intubation of adult for general anaesthesia	107	13	23% (3)
Administration of anaesthesia using bolus ketamine	336	10	110% (11)
Administration of anaesthesia using ketamine drip	93	10	30% (3)
Resuscitation of newborn	344	10	110% (11)

With the exception of one CEmOC trainee, all successfully passed both OSCE and written examinations which were conducted at the end of the training period. Written examinations for both programmes were composed and based on the format for national final qualifying examinations for the AMOs and included questions from topics that were considered as 'must know'. The OSCE for the CEmOC trainees was set to test the competencies to perform various important obstetric procedures which included vacuum deliveries, resuscitation of newborn babies and condom tamponade for management of postpartum haemorrhage.

The decision for either vaginal, operative vaginal or abdominal delivery was made by a team composed of all health care providers in the labour ward (midwives and doctors including the trainee). Individuals' ability for appropriate decision making for both training programmes were continuously assessed during the course and were at the end generally qualitatively judged to be satisfactory for all participants. The review team identified only one case with a major complication (severe postpartum haemorrhage) out of all procedures performed by the CEmOC trainees. This was judged to be due to retained products of conception after caesarean section. There was no mortality, sepsis, burst abdomen or any anaesthetic complications out of the cases performed by the trainees during the training period.

### Performances in the health centres

**Figure 1:**
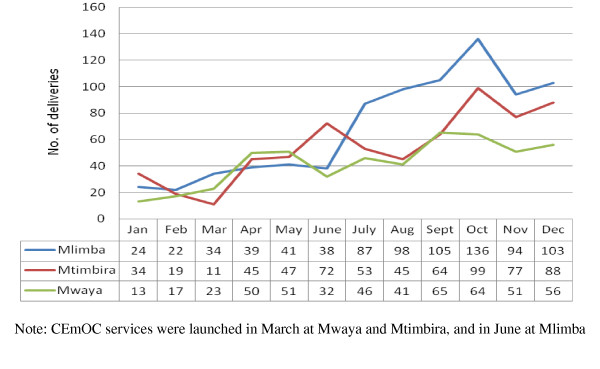
**The trend in monthly deliveries before and after launching CEmOC services in 2010 in the three remote health centres in Morogoro region, Tanzania**.

**Figure 2:**
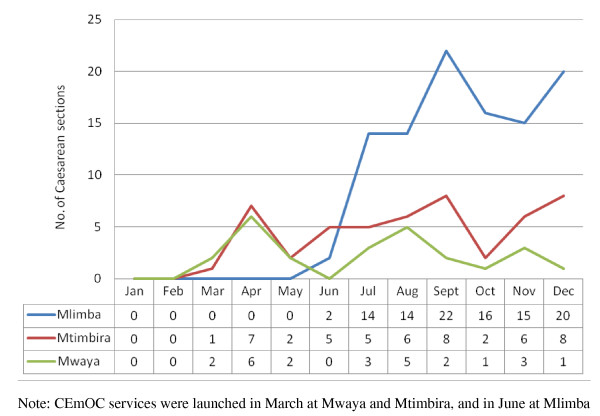
**The trend in monthly Caesarean section deliveries after introducing CEmOC services in 2010 in the three remote health centres in Morogoro region, Tanzania**.

Following introduction of CEmOC services the trends of total deliveries and caesarean sections increased remarkably in all three health centres, Mlimba, Mtimbira and Mwaya (see Figure 1 and 2). On average, monthly deliveries increased by as much as 300% at Mlimba health centre. Mtimbira and Mwaya health centres had less dramatic increases: these centres had had only one AMO each and the number of caesarean deliveries decreased whenever these AMOs were absent from their stations because of other obligations, illness, or training sessions required by the district administration.

Two maternal deaths were reported in two upgraded health centres (Mwaya and Mtimbira) after CEmOC services were introduced. These deaths were due to severe postpartum haemorrhage and puerperal sepsis following prolonged obstructed labour at home. Although statistically not significant fresh stillbirth rates declined by 60% after introduction of CEmOC services (July to December 2010) despite increased institutional deliveries (OR = 0.4; 95% CI: 0.1-1.7) compared to before (January-February). The number of referred obstetric cases declined significantly after introduction of CEmOC services (OR = 0.2; 95% CI: 0.1- 0.4) (Table 3).

**Table 3:** Proportions of fresh stillbirth and obstetric referrals before and after introducing CEmOC services in 2010 in three remote health centres in Morogoro region, Tanzania.

	Before CEmOC services (Jan-Feb	After CEmOC services (July-Dec)	OR (95% CI)
Fresh stillbirths			
Mtimbira	4	5	
Mlimba	0	4	
Mwaya	0	2	
Total SBF/total births	4/202	11/1372	0.4 (0.1-1.7)
Fresh stillbirth rate/1000 births	20	8	
Obstetric referrals			
Mtimbira	3	14	
Mlimba	5	3	
Mwaya	9	8	
Total	17	25	
Referral rate	8%	2%	0.2 (0.1-0.4)

Note: CEmOC services were launched in March 2010 at Mwaya and Mtimbira, and in June at Mlimba

## Discussion

Strengthening human resources for health is a central denominator for combating health crises and building sustainable health systems in resource limited countries [9-11]. The training of NPCs in Tanzania for maternal health care is one of the regional innovations based on local realities of high maternal and perinatal deaths and low met needs linked to severe shortage of qualified staff. The initiative applied the concept of 'task shifting' which has been advocated and proved useful for maternal health care in sub-Saharan Africa, where severe depletion of qualified staff exists [6,12]. These findings indicate that such training programmes can improve the knowledge and clinical management skills of NPCs and may subsequently improve the quality of maternal health care [13,14]. Considering that at least 5% of all pregnant women experience life-threatening complications possibly requiring caesarean section, and therefore anaesthesia, and the fact that tens of thousands of women die every year because of lack of these services [12,15], the training was crucial and may contribute to reduction of maternal and perinatal mortality and morbidity in 11 beneficiary districts with a total population of 2.6 million people [16].

Deeming the quality of the performances of these NPCs as acceptable following introduction of CEmOC services in the upgraded health centres is suggested by: the presence of only one severe complication out of 278 major obstetric surgeries and 1161 anaesthetic procedures performed during training; the small number of maternal deaths; and a reduced fresh stillbirth rate. Similar findings, regarding the quality of care and outcomes for major obstetric surgeries performed by NPCs, have been reported from within and outside the country and are comparable to those performed by graduate medical officers [6,17-20]. The increase of deliveries and caesarean sections in these health centres suggests improved accessibility to CEmOC services and possibly also improved pregnancy outcomes in the catchment areas.

The process for selecting trainees took into consideration the geographic distribution of the health facilities, an important UN process indicator for EmOC services [3]. Upgrading these facilities to provide CEmOC will significantly shorten the time wasted when referring women with obstetric complications. Successful reduction of maternal mortality in resource limited countries (such as Bangladesh, Bolivia and Honduras) has been linked to improved accessibility to health facility delivery services as well as improved quality of care during pregnancy, labour and the period immediately after birth [21,22]. These countries strategically targeted remote rural areas with high ratios of maternal mortality. This innovation calls for the global community to consider scaling up training and use of teams of NPCs for CEmOC and anaesthesia.

### Limitations of the training

Trainees had limited exposure to certain important obstetric and anaesthetic procedures, including vacuum delivery, surgeries for ruptured uterus and intubation for general anaesthesia. This could have been contributed by large groups of participants. Intubations for general anaesthesia were limited because of the costs involved for the drugs as compared to those for spinal anaesthesia. In an attempt to bridge these gaps, participants were also trained using models (available in clinical skills laboratory) for vacuum extraction and intubation. The authors also recommended technical support at the beginning and regular supportive supervision afterwards by more experienced staff. While still gaining confidence, trainees were advised to start with obstetric surgeries which are considered to be uncomplicated, such as straight forward caesarean section, and continue to refer complicated ones.

## Conclusions

Our findings indicate that health centres can be upgraded and NPCs trained to provide comprehensive EmOC. Considering that most Sub-Saharan countries are already off-track in their attempts to achieve the MDGs for maternal and perinatal survival, evidence resulting from the current training programmes calls for urgency to scale up the application of the concept of 'task shifting' with the use of NPCs for CEmOC services provision and anaesthesia.

## List of abbreviations

AMO: assistant medical officer; CEmOC: comprehensive emergency obstetric care; CO: clinical officer; MDG: Millennium Development Goals; NM: nurse-midwife; NPCs: Non-physician clinicians; OSCE: objectively structured clinical examination; SFDDH: Saint Francis Designated District Hospital; TTCIH: Tanzanian Training Centre for International Health; UNFPA: The United Nations Population Fund.

## Competing interests

The authors declare that they have no competing interests.

## Authors' contributions

ASN participated in curriculum development and implementation, data collection, analysis and wrote the manuscript. SKP participated in curriculum development and implementation and wrote the manuscript. GM reviewed the curriculum and contributed in manuscript writing. FDR contributed in curriculum implementation and reviewed the manuscript. JvR contributed in curriculum implementation and reviewed the manuscript. All authors read and approved the final manuscript.
